# Nativity differentials in first births in the United States: Patterns by race and ethnicity

**DOI:** 10.4054/demres.2022.46.2

**Published:** 2022-01-05

**Authors:** Andrés F. Castro Torres, Emilio Alberto Parrado

**Affiliations:** 1 Laboratory of Fertility and Well-being, Max-Planck-Institut für Demografische Forschung, Rostock, Germany.; 2 Department of Sociology, University of Pennsylvania, Philadelphia, USA.

## Abstract

**BACKGROUND:**

While recent decades have seen gradual convergence in ethno-racial disparities in completed fertility in the United States, differences in the age pattern of first births remain. The role of nativity has not been fully understood.

**OBJECTIVE:**

This paper examines how first births vary by nativity, and how this variation contributes to more significant racial and ethnic differentials.

**METHODS:**

Using data from the National Survey of Family Growth (1997–2017), we jointly estimate the correlates of the timing of first births and childlessness. We assess differences between immigrants and US-born and child-migrant women across ethno-racial groups.

**RESULTS:**

The unique first-birth patterns among foreign-born women have a notable impact on Hispanics, reducing differences from Whites in the average age at first birth and contributing to more significant differentials in childlessness. The impact of immigrant women on White and Black first births is more modest in scope.

**CONTRIBUTION:**

Our work shows the importance of nativity for ethnic/racial disparities in the timing and quantum of fertility in the United States. We demonstrate how the migrant population is more determinant for Hispanic fertility patterns than for Black or White. We conclude by elaborating on the implications of these results for future research as the immigrant population in the United States becomes ethnically and racially more diverse.

## 1. Introduction

A long tradition of migration studies investigates the link between international migration and various dimensions of fertility behavior. The most common finding within this scholarship is that immigrant women’s fertility patterns differ from those observed among non-migrant women in both sending and receiving contexts (Adserà and Ferrer 2015; Clark, Glick, and Bures 2009; Zárate and Unger De Zárate 1975). However, most studies tend to focus on overall fertility rates or family size, to the relative neglect of other aspects of childbearing (Bagavos 2019; Bernard, Bell, and Charles-Edwards 2014; Parrado 2011). The pattern of first births is a case in point, despite notable exceptions (Choi 2014; Goldberg 2018; Guarini et al. 2015). The transition to parenthood is a major life-course event that impacts multiple other domains, from union formation and dissolution to labor market and residential patterns (Hogan 1978; Rindfuss, Morgan, and Swicegoog 1988). The prevalence of first-birth transitions also has implications for discussions around childlessness (Rindfuss, Morgan, and Offutt 1996; van Rooij, van Balen, and Hermanns 2009; Sobotka 2017; Stone 2014). Whether and how the timing and prevalence of first births are shaped by nativity is thus an important question.

Nativity differentials in the timing and prevalence of first births are particularly relevant in the United States, given the impact of immigration on the US population (Frank and Heuveline 2005; Livingston 2016; Sobotka 2008). Between 1980 and 2015 the foreign-born share of women of reproductive ages (14–49) grew 2.6 times, from 7% to 18%. However, there is dramatic variation across ethno-racial groups. According to data from the American Community Survey, in 2017 the share of foreign-born was highest for Asia women (75%), followed by Hispanic (39%), Black (12%), and White women (5%).

Nativity differentials have direct implications for understanding racial and ethnic disparities in fertility (Camarota and Zeigler 2019; Carlson 1985; Parrado 2015; Schoorl 1990). Despite recent convergence in family size across racial and ethnic groups, sizeable disparities in the timing of fertility and prevalence of childlessness remain (Carter 2000; Landale and Oropesa 2007; Rauh, Andrews, and Garfinkel 2001; Yang and Morgan 2003). Sweeney and Raley (2014) document that the number of children born to women aged 40–44 (i.e., completed fertility) is roughly similar for White and Black women (around 1.8) and only slightly higher for Hispanic women (about 2.1). Cai and Morgan (2019) report lower complete fertility among women of Chinese, Japanese, and Korean origin (1.5 children for the 2000–2014 period). However, the age pattern of childbearing remains very uneven, with Black and Hispanic women averaging substantially younger ages at first birth and lower prevalence of childlessness than White women (Sullivan 2005), whereas women of Asian origin display higher ages of childbearing (Cai and Morgan 2019). The position of immigrant women within these trends is not well understood (Parrado and Flippen 2012; Parrado and Morgan 2008).

This paper investigates nativity differentials in first births in the United States and their variation by race and ethnicity. We focus on both first-birth timing and prevalence to separate nativity differentials in the age pattern of childbearing and childlessness. Data for the analysis come from five waves (1997–2017) of the National Survey of Family Growth (NSFG). Due to sample size limitations we cannot incorporate Asian women into the analysis and focus on non-Hispanic White (hereafter White), non-Hispanic Black (hereafter Black), and Hispanic differentials in first births. Therefore, our conclusions do not apply to Asian migrants, which is a limitation of our study given the particularity of fertility patterns among this group. However, their overall influence on aggregate first-birth patterns is limited given their relatively small size as group (among both foreign- and US-born), and their relative low fertility (Martin et al. 2017).

The results document that, net of socioeconomic background, immigrant women exhibit a later pattern of first births relative to their US-born and child-migrant ethno-racial counterparts (details about comparisons groups are in the Data and Methods section). The experience is common to all immigrant groups. However, unlike the pattern in the general population, the results indicate that delayed first births do not correspond with a higher prevalence of childlessness among immigrants. We highlight the importance of separating immigrant women for understanding racial and ethnic disparities in first births, especially for groups with large and growing immigrant representation, and discuss the implications of immigration for ethno-racial differences in fertility. Consistent with previous formulations of the role of migration on population dynamics, our results show that the influence of migrant populations on the timing of first births across the US ethno-racial groups depends on the size of the migrant population, the duration of migration (stayers vs. returners), and the socioeconomic background of the migrants (Adserà and Ferrer 2014; Portes 2010).

## 2. Theoretical background: Linking ethno-racial and nativity disparities in first births

Since the 1970s, two related trends in first-birth patterns have been documented in the United States. The first is the considerable delay in the transition to parenthood, accompanied by increased fertility at older ages (Hayford, Guzzo, and Smock 2014; Martin 2000; Sobotka 2017). Second, fertility postponement has resulted not only in older ages at first birth but also in an increasing proportion of women remaining childless at the end of their reproductive years (Chen and Morgan 1991). However, these patterns more closely fit the experience of White than non-White women. Despite considerable declines in teenage childbearing, Black and Hispanic women continue to exhibit younger ages at first birth than Whites: in 2012 the mean age at first birth was 23.6 and 23.8 for Black and Hispanic women respectively, compared to 26.6 for White women (Sweeney and Raley 2014). Similarly, while delayed childbearing resulted in a higher proportion of women remaining childless for all groups, the prevalence is much higher among Whites. Estimates indicate that in 2010 the share of childless at ages 40 to 44 was 21% among White women compared to 17% and 12% among Black and Hispanic women, respectively (Sweeney and Raley 2014).

Three general perspectives are useful for understanding the connection between immigration and first births (Bean, Swicegood, and Berg 2000; Bohon and Conley 2015; Gorwaney et al. 1990; Hervitz 1985; Massey and Mullan 1984; Stephen and Bean 1992). The selection perspective expects the fertility of immigrants to differ from that of the native-born population because the former are selected according to socioeconomic characteristics that are also associated with fertility, such as level of education (Adserà and Ferrer 2014; Jensen and Ahlburg 2004; Lindstrom, Hernandez-Jabalera, and Giorguli Saucedo 2021; Mussino and Strozza 2012). The direction of the disparity is dependent on the nature of selection into migration. For positively selected immigrant flows, such as White (predominantly European) and Black (mainly African and Caribbean) immigrants to the United States, above-average educational attainment and socioeconomic background could result in later ages at first birth relative to US-born women. The opposite can be expected for immigrant flows with relatively low levels of educational attainment, such as those from Latin America, where the foreign-born could be expected to average younger ages at first birth than their native-born counterparts (Rendall and Parker 2014). Overall, the general expectation from the selection perspective is that compositional differences between foreign- and native-born women undergird observed differences in fertility.

Similar theoretical considerations apply to the role of migrant selectivity on the prevalence of childlessness, although expectations in this respect are less clear (Nedoluzhko and Andersson 2007). It is conceivable that the inverse association between education and childlessness found among the general population is reproduced among immigrants. Thus, when immigrants have lower levels of education than their US coethnics, immigration will tend to reduce childlessness, while highly educated immigration flows will have the opposite effect.

The disruption perspective highlights that migration, especially when international, disrupts the life-course trajectories of individuals in a manner not experienced by non-migrants (Bach 1981; Lindstrom and Giorguli Saucedo 2002, 2007; Mussino, Wilson, and Andersson 2020). Migration often entails family separation, which might disrupt patterns of childbearing (Andersson, Obucina, and Scott 2015; Boyle et al. 2008; Chattopadhyay, White, and Debpuur 2006). Even in the absence of separation, the act of relocating to a foreign country can generate considerable uncertainty and short-term disruption to family plans. Like investments in other human capital dimensions, women might delay childbearing in anticipation of migration. It might also take women some time to rebuild childbearing expectations after arriving at their destination. The disruptive effect can also be connected to delayed marriage or union formation in the case of women migrating single (Lindstrom and Giorguli Saucedo 2002; Mussino, Wilson, and Andersson 2020). Thus, the general expectation from the disruption hypothesis is that net of socioeconomic background characteristics, international migration will be associated with postponed life-course transitions – including first births – among migrants relative to non-migrants, with scant variation across racial/ethnic groups. To the extent that disruption leads to postponed fertility, this perspective would expect a higher prevalence of childlessness among immigrants relative to their US-born counterparts. Again, the pattern is not expected to differ across racial/ethnic groups.

Finally, the life-course perspective stresses the link between life-course events, including migration and childbearing (Bernard, Bell, and Charles-Edwards 2014; Clark, Glick, and Bures 2009; Wilson 2020). Especially in the case of women’s migration, studies have documented a close connection between migration and family formation (Mulder and Wagner 1993), with immigrant women showing a tendency to form unions and have children shortly after migrating to the United States (Lindstrom, Hernandez-Jabalera, and Giorguli Saucedo 2021; Ortensi 2015; Parrado 2015). Studies have shown that immigrant women are more likely to be married than non-immigrant women of the same age, and the pattern is not exclusively the result of women migrating after marriage but also of rapid union formation after migration (Choi and Tienda 2017; Raley, Durden, and Wildsmith 2004). However, while the implications for the timing of childbearing do not differ from those obtained from the selection and disruption perspectives, the life-course perspective has direct implications for expectations about childlessness. To the extent that family formation and childbearing are an integral part of the decision to migrate and settle in the United States, we would expect the prevalence of childlessness to be lower among immigrant women than their non-migrant counterparts, irrespective of the educational composition of migration flows or the disruptive effect of migration.

## 3. Data and methods

We investigate nativity differentials in first births using data from seven waves (1995, 2002, 2006, 2008–2010, 2011–2013, 2013–2015, 2015–2017) of the National Survey of Family Growth (NSFG). These surveys are representative of the noninstitutionalized population of US women aged 15 to 44 – and since 2015 the age range has been extended to 49. The surveys contain comparable retrospective information on our main dependent variables, namely the prevalence and timing of first births. The data also includes information about our main correlates of interest, including race and ethnicity, nativity, and educational attainment. All these survey waves oversampled Hispanic, non-Hispanic Black, and teenage women, which makes them an ideal data source to compare first birth patterns by nativity among these two racial/ethnic groups, in addition to non-Hispanic Whites, the other (by default) big-sample-size racial/ethnic group.

Table 1 shows the total number of women interviewed by the NSFG from 1995 to 2015 by race/ethnicity. The last two rows of the table indicate the total sample size for the three racial/ethnic groups under study, and the size of the analytical sample after excluding records with missing information in the covariates (139 records). Out of the 44,946 women in the analytical sample 18,964 (42%) reported an age at first birth; all other records are treated as right-censored.

Despite their growing significance for the US population, the number of Asian women in the United States and their contribution to first births is small (e.g., 8.0% of first births in 2015 were to Asian or Pacific islander women, according to Martin et al. (2017)). Therefore, their share in nationally representative surveys such as the NSFG is limited. As reported in Table 1, there are only 2,538 women classified as ‘Other’ according to the racial/ethnic classification, which prevents us from analyzing racial/ethnic groups other than Hispanic, Black, and White.

Given our focus on fertility, foreign-born women are defined as those arriving in the United States after age 14 (i.e., teenage and adult migrants). Teenage and adult migrants were socialized in a foreign environment (outside of the United States), and their risk of childbearing is initiated before the time of arrival in the United States, which arguably makes them less similar to US-born women; indeed, some of these immigrant women could already be mothers at the time of their migration. The reverse is true for women arriving before age 14 (i.e., child migrants). The socialization and risk of childbearing for these women occurs almost entirely in the United States, which makes them more similar to the US-born population. Therefore, we group US-born women and child migrants into a single group (i.e., “US-born and child migrants”), and we test the robustness of our results by excluding child migrants from the analysis.

Our statistical specification follows our focus on nativity differentials in the timing and prevalence of first births and their variation by race/ethnicity. To jointly estimate the two dimensions while still allowing for their separate analysis, we formulate split-population duration models. Split-population models are a special kind of frailty model which, in addition to accounting for the right-censored nature of cross-sectional data, recognize that observations come from a population that splits between a group that is at risk of experiencing the event (in our case, first birth) and a group that is not. Conceptually, they overcome some of the ambiguities in standard duration models that do not separate the likelihood of occurrence of a first birth from the age at which it occurs (Gray et al. 2010; Johnson et al. 2018). Yamaguchi and Ferguson (1995) applied the methodology to separate the impact of gender preferences on the timing and prevalence of third births. In medical research, the approach is sometimes referred to as “cured” models because they recognize that not all individuals will experience a disease (Bremhorst, Kreyenfeld, and Lambert 2016). In the context of low fertility and rising childlessness, the two dimensions capture very different fertility processes. Separating timing from occurrence can be particularly important for adjudicating between selection, disruption, and life-course perspectives, as the two dimensions offer more variation in predictions regarding nativity and ethno-racial differentials in childbearing.

Formally, the split-population duration models recognize that the survival function has a limit probability value, π, rather than 0. There are two types of split-population models depending on how the survival function is specified: mixture and non-mixture (Gray et al. 2010). In the mixture model, the survival function is expressed as:

$$S(t)=\pi +(1-\pi )S_{u}(t)$$

where *S(t)* is the probability of not having a birth by age *t*, π is the proportion not having a birth by age 45, and *Su(t)* is the survival function for women having children.

In the non-mixture model, the survival function becomes:

$$S(t)=\pi^{Fz(t)}$$

where π is also the proportion not having a birth by age 45 and *Fz(t)* is the cumulative distribution function of the progression to first birth. Substantively, they differ in their assumptions regarding the proportion not exposed to the event. The mixture model assumes that a proportion of women will remain childless at the beginning of the observation period. The non-mixture model assumes a cumulative distribution function with values that range from 0 to 1 at the beginning of the observation period, so all women can have a child, but as time passes the eventual survival fraction reaches *π*. Arguments can be made in favor of each of the models; we compare results from both specifications using goodness-of-fit measures. Both specifications allow for different parametric distributions of the survival function, such as exponential, Weibull, lognormal, or gamma. As in prior fertility analyses (Gray et al. 2010:201; Varga 2014), goodness-of-fit tests were generally better for non-mixture models with a lognormal distribution with survivor function *S(t)* = 1-ϕ {(log(*t_j_*) - μ_*j*_)/σ} where ϕ is the standard normal cumulative function. Comparisons with alternative distributions show that results do not vary substantively across specifications.

A split-population formulation allows us to jointly model the effect of individual predictors on the proportion of women remaining childless and the timing of first births for women who do eventually have a child. The cure fraction *π* is modeled with a logistic link function where *π* = *exp(xβ)/(1*+*exp(xβ))*. The lognormal regression estimating the timing of childbearing is implemented by setting the mean age of first birth or scale parameter to μ_*j*_ = *exp(x_j_β)* and the standard deviation or shape parameter to σ = *exp(x_j_β)*.

In addition to race/ethnicity and nativity, the model includes educational attainment, survey year, and birth cohort as predictors. Educational attainment is captured with a set of mutually exclusive dummy variables indexing whether a woman has less than 9 (reference), 10–12, 13–15, or 16 or more years of education. In all models we include controls for survey year to capture sample variations (including the higher age of observation for the 2015 sample), as well as controls for cohort, since retrospective information on conceptions could be affected by a woman’s age at interview. Birth cohort is introduced as a set of dummy indicators indexing 10-year birth cohorts starting in 1950.

## 4. Descriptive results

### 4.1 First-birth timing and prevalence

**Figure 1:**
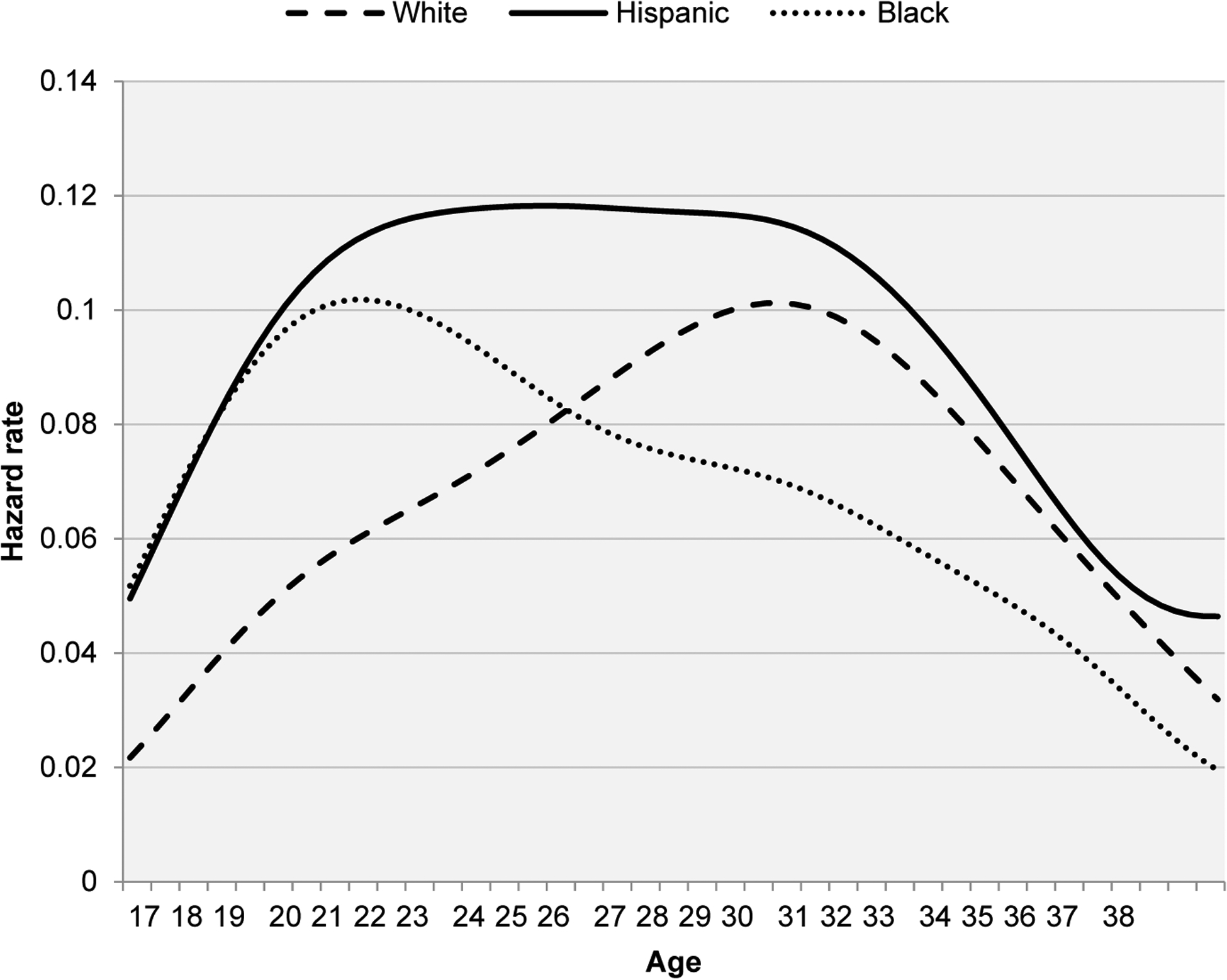
Racial-ethnic differentials in first-birth hazard rates by age *Note*: Results are obtained using sampling weights for pooled waves of the National Survey of Family Growth from 1995 to 2017 (n = 44,946 women). Data available at: https://www.cdc.gov/nchs/nsfg/index.htm.

Figure 1 plots the hazard rate of first birth by age and race/ethnicity. The age-pattern of childbearing for White women is consistent with a pattern of late fertility. The hazard rises slowly with age, peaks in the late 20s–early 30s, and then rapidly declines. The curve for Blacks depicts a pattern of early childbearing with a peak in the mid-20s followed by a rapid decline. For Hispanics, in turn, the hazard rises rapidly at early ages, remains high throughout the mid-30s, and then declines. The patterns result in considerable differences in the likelihood of experiencing a first birth by age 45. Estimates from the survival function show that 18.2%, 17.3%, and 9.6% of White, Black, and Hispanic women respectively will remain childless by age 45.

**Figure 2:**
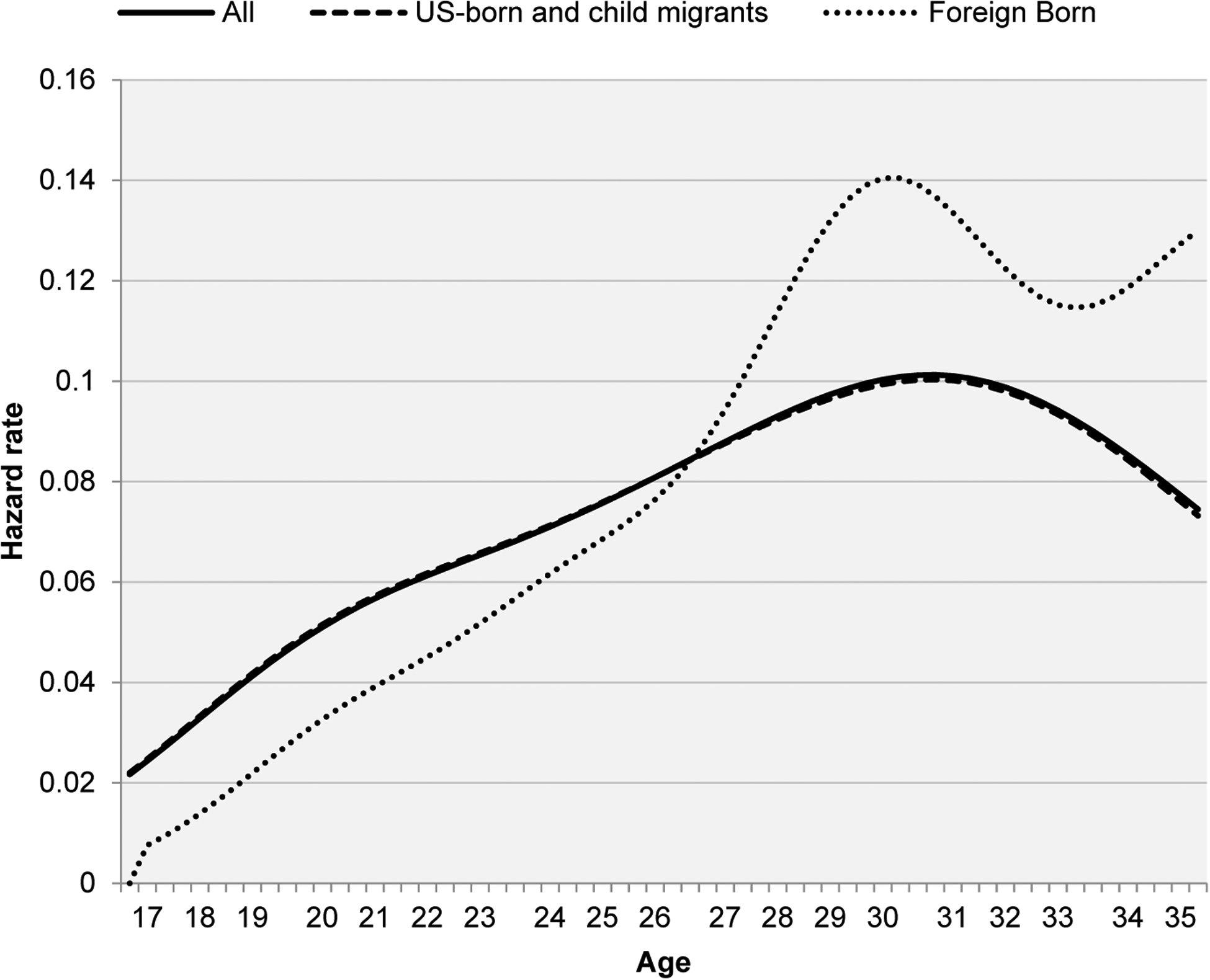
Nativity differentials in first-birth hazard rates by age – White women *Note*: Results are obtained using sampling weights for pooled waves of the National Survey of Family Growth from 1995 to 2017 (n = 24,848 women). Data available at: https://www.cdc.gov/nchs/nsfg/index.htm

**Figure 3:**
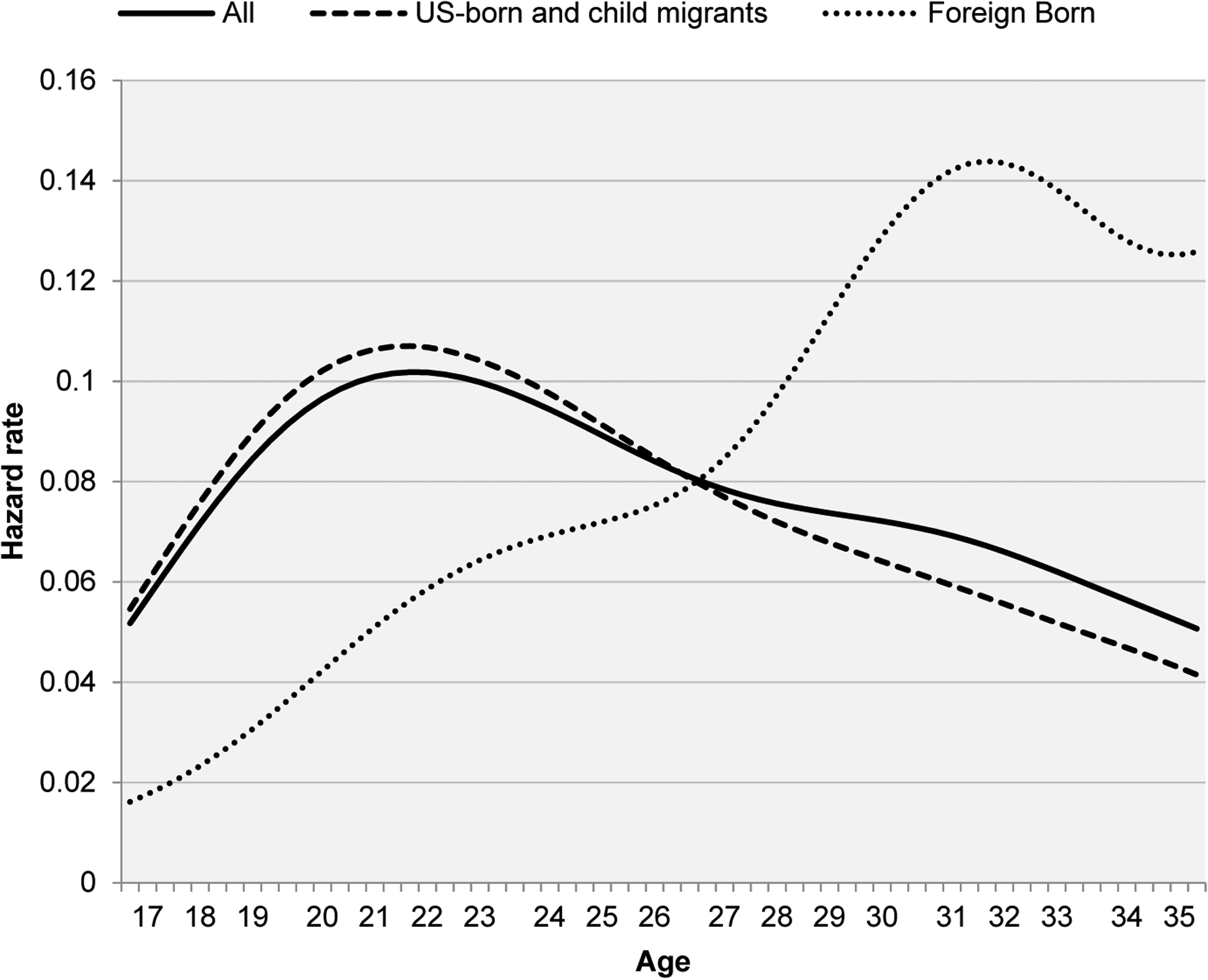
Nativity differentials in first-birth hazard rates by age – Black women *Note*: Results are obtained using sampling weights for pooled waves of the National Survey of Family Growth from 1995 to 2017 (n = 10,189 women). Data available at: https://www.cdc.gov/nchs/nsfg/index.htm

**Figure 4:**
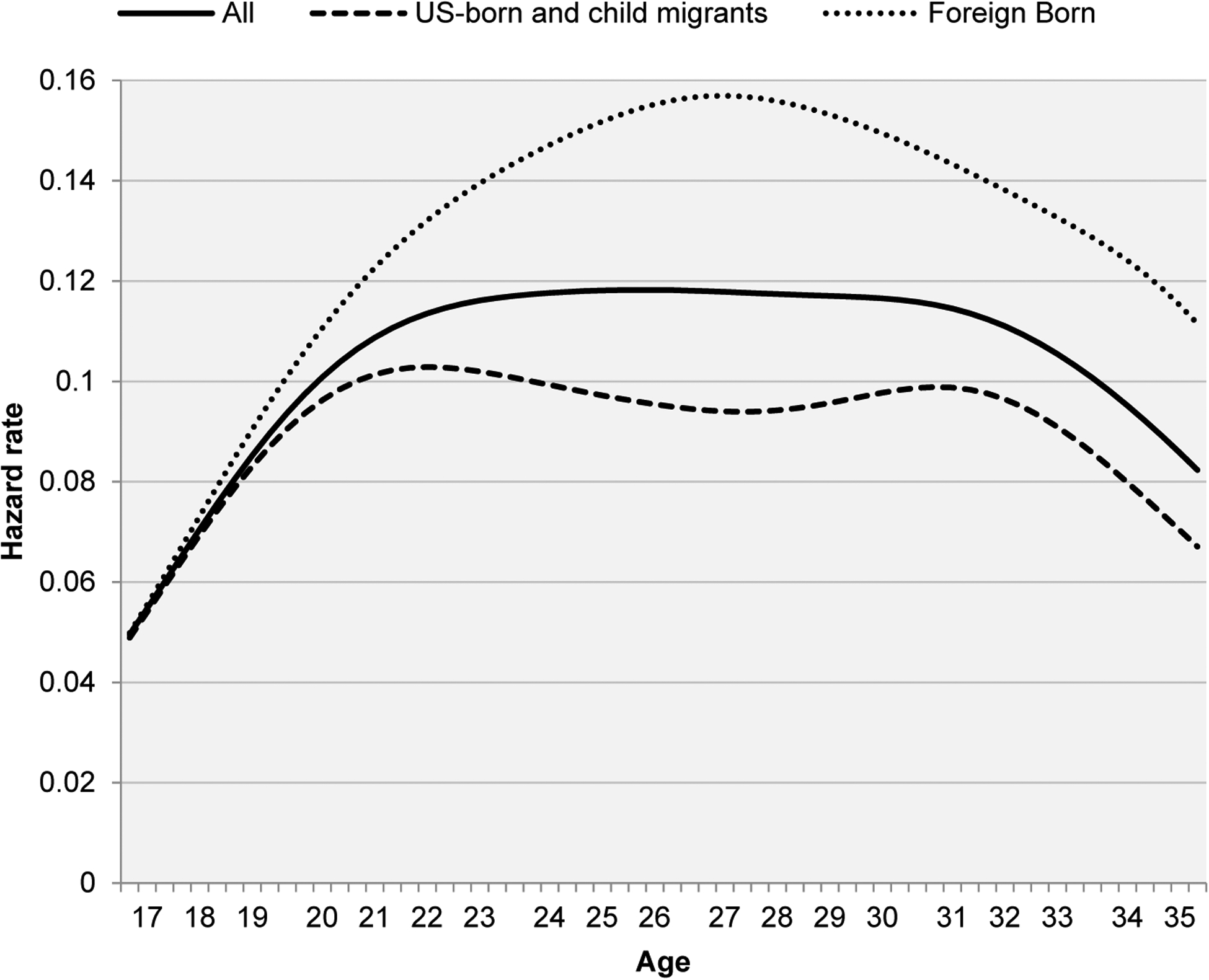
Nativity differentials in first-birth hazard rates by age – Hispanic women *Note*: Results are obtained using sampling weights for pooled waves of the National Survey of Family Growth from 1995 to 2017 (n = 9,909 women). Data available at: https://www.cdc.gov/nchs/nsfg/index.htm

Figures 2, 3, and 4 show first-birth rates for White, Black, and Hispanic women respectively, separating US-born and child migrants from foreign-born arriving after age 14 (Foreign-born herein). The results confirm substantial differences in first-birth patterns by nativity, with important variation across racial and ethnic groups. The results for White women (Figure 2) document considerable delays in the age at first birth among foreign-born relative to native-born and child migrant women. Foreign-born women exhibit a much lower risk of first birth at younger ages, before age 26, but a much higher risk afterward. Estimates from the survival function show that the median age at first child is two years older for immigrants than for native and child-migrant women (29 vs. 27 years of age). It is important to note that only 4% of first births among Whites were to foreign-born women, so the impact of the nativity difference on the White experience overall is negligible, as documented by the overlapping hazard rates between native-born and all White women in Figure 2. The difference also results in sizeable disparities in childlessness. By age 45, 18.5% of US-born and child-migrant women are childless, compared to a much lower 10.5% among foreign-born White women.

Among Black women (Figure 3), immigrants also exhibit later ages at first birth relative to their US-born and child-migrant counterparts. The gap is even more pronounced than among White women due to the relatively young childbearing pattern exhibited by US-born and child-migrant Black women. The median age at first birth among native-born women is 23 years, five years younger than the median age for immigrants (28). Because immigrants represent a larger share of women of reproductive ages among Blacks (8%) the divergent timing impacts the childbearing pattern of the group, reducing the prevalence of first births at young ages and increasing it at later ages. As was the case for White women, foreign-born Blacks have a much lower prevalence of remaining childless by age 45: 10.2%, compared to 18.5% among the US-born and child migrants.

A different pattern is evidenced for Hispanic women (Figure 4). The results document that the hazard rate of a first birth is dramatically higher among the foreign-born than for native-born and child-migrant women at all ages. As a result, the median age at first child is close to one year younger among the foreign-born than the native-born and child-migrant Hispanic women (22 vs. 23 years of age). US-born and child-migrant Hispanic women exhibit a bimodal age pattern of first births that peaks at early ages before age 22, then declines, and increases again after age 30. The much higher rate for the foreign-born at all ages results in a flat pattern of relatively high risk of first birth for all Hispanic women that spans ages 22 to 31. The pattern also translates into considerable differences in childlessness, with 13.1% of native and child-migrant women and 5.5% of foreign Hispanic women not having children by age 45.

### 4.2 Compositional differences between foreign- and US-born women by race/ethnicity

Appendix 1 reports descriptive statistics for the variables included in the multivariate model. Overall, 7.9% of our sample is foreign-born, with substantial variation across groups. Only 2.2% of White women are foreign-born, compared to a much higher 6.4% among Black women and a dramatically higher 29.9% among Hispanics.

Differences in educational attainment across groups highlight the importance of selection into migration. Among Whites, results document substantial positive selectivity in the migrant flow. While 31% of US-born and child-migrant White women have 16 or more years of education, the representation is 52.7% among the foreign-born. Apparent positive immigrant selectivity is also evidenced among Black women. The share with 16 or more years of education among Black immigrants (33.3%) is more than double the comparable figure for native-born and child-migrant Black women (15.8%). However, it remains lower than that evidenced by White immigrant women. Hispanics, by contrast, are the only group where the share with 16 or more years of education is lower for immigrants than native-born and child-migrant women (11.6% vs. 13.4%). They are also far more likely than their US-born and child-migrant peers to be among the least-educated category, with nine or fewer years of completed education (44.4% vs. 14.4 %). Given these sizeable differences in key predictors of childbearing, it is important to examine nativity and ethno-racial differentials in first births in a multivariate context.

## 5. Multivariate results

Table 2 reports coefficients from split-population log normal duration models jointly predicting the timing and prevalence of first births (estimates for survey year and cohort controls are included but not reported). Table 3 reports predicted estimates of the conditional mean age at first birth and prevalence of childlessness, according to average socioeconomic characteristics, for the whole sample by race/ethnicity and nativity, to assist in the interpretation of the coefficients. In each table, the top panel reports results from models predicting age at first birth, while the bottom panel reports results from models predicting childlessness at age 45.

We focus first on the timing of first births (top panel Tables 2 and 3). Model 1 shows that significant racial/ethnic differences remain even after jointly estimating timing and prevalence. According to Table 2, the coefficients for Black (−0.125) and Hispanic (−0.071) women imply faster transition to motherhood compared to White women. Predicted results show that the average conditional age at first birth is 27.6, 24.3, and 25.7 for White, Black, and Hispanic women, respectively (Table 3). Adding a control for nativity in Model 2 confirms that immigrant women average considerably later ages at first birth (coefficient = 0.08, s.e. = 0.007, predicted mean age at first birth = 28.3 years) than US-born women. While controlling for nativity (comparing Models 1 and 2 in Table 3) does not affect estimates of age at first birth for White and Black women (27.6 vs. 27.5 for White women and 24.3 vs. 24.2 for Black women), it reduces the age at first birth among Hispanic women by almost one year, from 25.7 to 24.8. In fact, the nativity differential for Hispanic women is large enough to affect overall racial and ethnic inequalities in first-birth timing. While in Model 1 Hispanics had a predicted average age at first birth that was 1.9 years younger than that of White women (25.7 vs. 27.6), the difference increases to 2.7 years (24.8 vs. 27.5) when comparing native-born and child-migrant women in Model 2. The opposite change is evident for the difference between Black and Hispanic women. While the predicted average age at first birth was 1.4 years older among Hispanic than Black women in Model 1, the difference is only 0.6 years when we focus on US-born and child-migrant women in Model 2. Across all these models, the uncertainty of the racial/ethnic coefficients is very low (s.e. ≤ 0.005), which warrants the comparison of predicted conditional outcomes across racial/ethnic groups.

Model 3 introduces an interaction term between foreign-born status and race/ethnicity. Results show that the pattern of later ages at first birth among immigrants is observed for all groups. However, there is variation in the distance separating US-born and child migrants, and foreign-born women by race/ethnicity. Among Whites, the foreign-born have a conditional age at first birth that is 1.156 (exp(0.145)) times later than US-born and child-migrant women, according to the coefficients reported in Table 2. Likewise, the age difference is higher among Blacks, among whom immigrants’ age at first birth averages 1.215 (exp(0.145+0.049) times higher than among native-born and child-migrant Black women. On the other hand, among Hispanics the difference is far smaller, even though immigrants continue to exhibit later ages at first birth than US-born and child-migrant women. Immigrant women’s age at first birth is only 1.057 (exp(0.145+(−.0090)) times higher than their native-born and child-migrant Hispanic peers, according to the coefficients for Model 3 (Table 2). The standard errors of the interaction coefficients are low relative to the coefficients themselves (except for Black women due to small sample size among foreign-born), which allows us to examine differences in mean ages at first birth by race/ethnicity and nativity. Predicted estimates in Table 3 illustrate these differences. Foreign-born White women have an average conditional age at first birth that is 4.3 years older than US-born and child-migrant White women, compared to 5.2 years among Black women and only 1.4 years among Hispanic women.

Model 4 adds control for educational attainment. As expected, more-educated women average later ages at first birth than their less-educated counterparts. Accounting for education reduces but does not eliminate racial/ethnic differentials in first births among US-born and child-migrant women. After educational disparities across groups are taken into account, Black and Hispanic women average 0.906 (exp(−0.099)) and 0.949 (exp(−0.053)) times younger ages at first birth than White women respectively.

Accounting for the educational selectivity of immigrant flows accounts for a large share of the nativity differential in the timing of first births. Notably, standard errors for the interaction term coefficients in Model 4 are slightly lower than those yielded by Model 3, which signal a better fit once educational attainment is used as predictor. Among White women the results show that the much later ages at first birth among immigrants relative to natives and child migrants, identified in Model 3, are primarily the outcome of the positive educational selectivity of immigrant women. For White women, the later average age at first birth is reduced from 27.4 in Model 3 to 26.6 in Model 4 (Table 2). Before controlling for educational attainment, the conditional mean age at first birth for the average White immigrant is 31.7 years. Accounting for education reduces the age at first birth by 2.7 years, to 29.0. While this is still later than the predicted 26.6 years of age for the average native-born or child-migrant White woman, the gap is far less pronounced after accounting for compositional differences across groups.

Accounting for education also reduces the age gap at first birth between US-born and child migrants and foreign-born Black women (1.161 times (exp(0.088+0.061)), although the change between Models 3 and 4 is less pronounced than among White women. Like White immigrants, Black immigrant women average higher educational attainment than their US-born and child-migrant counterparts. This positive selectivity partially accounts for their later ages at first birth. Controlling for education reduces the conditional age at first birth for Black immigrants from 29.2 to 28.0 years, reducing the age disparity relative to their US-born and child-migrant counterparts from 5.2 to 3.9 years.

Accounting for educational disparities between foreign-born women on the one hand and native-born and child-migrant women on the other has an even more striking impact on first-birth differentials among Hispanic women. Hispanic immigrants average lower educational attainment than their US-born and child-migrant peers and other immigrant groups. As such, while accounting for education reduces the nativity differential in age at first birth among White and Black women, the opposite is true for Hispanics. If immigrant Hispanics had the same educational composition as their native-born counterparts, their average age at first birth would be more than a year higher (27.6 vs. 26.5) and the disparity with US-born and child-migrant women would be even larger (2.4 vs. 1.4 years). In addition, the younger ages at first birth among Hispanic immigrants relative to foreign-born White women documented in Model 3 is entirely explained by the educational composition of the groups (Model 4). Accounting for education increases the conditional age at first birth among Hispanic immigrants by over a year, from 26.5 to 27.6 years. The result is a growing disparity in age at first birth between Hispanic immigrants and native and child migrants, from 1.4 to 2.4 years (Model 3 vs. Model 4).

Thus, overall, the results document the salience of educational selectivity for understanding first-birth patterns among immigrant women and differences with their US-born and child-migrant ethno-racial counterparts. However, they also show that the disruptive effect of migration is present among all immigrant groups, irrespective of racial/ethnic background.

The bottom panels of Tables 2 and 3 present logistic regression estimates of the likelihood of remaining childless by age 45 and average predicted probabilities, respectively. Model 1 documents that Black and Hispanic women are less likely to remain childless than White women. The difference between Hispanic and White women is particularly pronounced (coefficients −0.335 vs. −0.821). Predicted probabilities in Table 2 show that at age 45, 17.3% of White women are childless, compared to 13.0% and 8.4% for Black and Hispanic women, respectively. Adding a control for foreign-born status in Model 2 shows that immigrants are 0.527 (exp(−0.6410)) times as likely to remain childless as US-born and child-migrant women, with the predicted prevalence of childlessness among the foreign-born only 9.5%. As a result, separating the foreign-born results in a higher likelihood of childlessness among native-born and child-migrant women of 19.5% among Whites, 14.9% among Blacks, and 12.3% among Hispanics. More importantly, given the high immigrant representation among Hispanics, the change is particularly pronounced among that group. Separating immigrants from native-born and child-migrant women also reduces ethno-racial disparities in childlessness.

Model 3 interacts nativity with race/ethnicity. Contrary to results obtained for the timing of first birth, the lower likelihood of remaining childless among immigrants relative to their native-born and child-migrant counterparts is statistically equivalent for all ethno-racial groups. Model 4, which adds controls for educational background, confirms that the likelihood of remaining childless increases with higher levels of educational attainment. However, in another departure from the pattern for age at first birth, differences in the educational composition of immigrant v. native-born and child-migrant women does not explain disparities in childlessness across groups. After controlling for education and separating immigrants according to race and ethnicity, the predicted prevalence of childlessness is roughly the same for all groups, around 8%. This finding is consistent with the perspective that stresses that immigration and family formation are tightly interwoven life-course transitions irrespective of ethno-racial origin.

Finally, it is worth mentioning that these results are robust to the exclusion of child migrants, meaning that the racial/ethnic disparities and disruptive role of the migration experience are also valid when the comparison group is strictly the US-born population.

## 6. Conclusions

This paper investigates the role of immigration in shaping racial and ethnic differences in first births. Using data from the NSFG spanning 1995 through 2017, our analysis focuses on two distinct dimensions of first births: timing and prevalence. We formulate split-population models that explicitly separate the study of age at first birth from childlessness and systematically compare the experiences of foreign-born, on the one hand, and native-born and child-migrant White, Black, and Hispanic women on the other. This comparison allows for evaluating perspectives on immigrant childbearing centered on selection, disruption, and the connection between life-cycle events, with important implications for understanding ethno-racial differentials in family behavior in the United States.

The results support expectations from disruption perspectives that frame migration as an event that alters the fertility trajectories of women. Immigrants consistently exhibit noticeably later ages at first birth than US-born and child-migrant women of the same racial/ethnic group, which in in line with previous studies on immigrants/adolescent immigrants in the United States (Goldberg 2018; Guarini et al. 2015). Our paper provides updated and nationally representative estimates of the delayed transitions to first birth associated with the migration experience. However, unlike the pattern evident in the general population, among immigrants a higher average age at first birth does not translate into a higher proportion childless at age 45. Instead, immigrant women are considerably less likely than their native-born and child-migrant counterparts to remain childless, a pattern consistent with life-cycle perspectives that view migration and family formation as intimately interconnected events. This pattern does not vary across ethno-racial groups, despite substantial differences in region of origin and pattern of selectivity into migration, suggesting that the link between migration and family formation is a common experience among immigrant women, at least for those who stayed in the United States.

We also show that differences among immigrant women in the timing and prevalence of first birth can be large enough to affect overall ethno-racial differential childbearing patterns. This is an important contribution, because previous studies either focus on Mexican/Hispanics or do not examine the implications of nativity for racial/ethnic groups (Choi 2014; Goldberg 2018; Guarini et al. 2015). Distinguishing between native-born and child migrants and foreign-born childbearing patterns increases the White–Black and White–Hispanic differential in age at first birth among US-born women by 2.5 and 1.4 years, respectively. The opposite result is obtained for the prevalence of childlessness. The substantially higher prevalence of childlessness among White women compared to Black and Hispanic women is markedly reduced when we focus exclusively on differentials among the native-born. Thus, failing to account for the unique childbearing profile of immigrant women masks ethno-racial variation in age at first birth while exaggerating differences in childlessness across groups.

The selectivity of the immigrant flow partly accounts for differences across ethno-racial groups. Much of the disparity between foreign-born White and Hispanic women in timing and prevalence of first birth stems from the much lower educational attainment of Hispanic immigrants, a well-documented fact in the literature that further contributes to the delayed transition to childbearing and marriage among immigrants (Glick et al. 2006). However, the opposite is true for Black immigrants, who average high levels of educational attainment.

Overall, the analysis highlights the salient role of immigration in understanding racial and ethnic disparities in fertility. To the extent that immigrant representation continues to grow, and especially given the considerable variation in immigrant representation within racial and ethnic groups, it has become increasingly imperative to separate immigrants when assessing racial and ethnic differentials in fertility. This is particularly important for Hispanic women, among whom a high share is foreign-born, with patterns of educational attainment that depart from those of the receiving population. The literature on ethno-racial differences in family behavior often frames Hispanic women as outliers, drawing on cultural explanations and framing persistent differences between native-born Hispanic and White women as evidence of a lack of cultural assimilation.

A main implication is that attempts to diagnose and promote reproductive health behaviors leading to reduction in early and unplanned childbearing, especially among minorities, need to recognize the unique position of immigrant women relative to their US-born counterparts. There is increasing recognition of the considerable heterogeneity within racial and ethnic groups in terms of socioeconomic background and family experience. Our analysis highlights foreign-born status as an additional source of heterogeneity that is not always recognized or addressed in the formulation of reproductive health policies. Therefore, in both future research and family-related policymaking it is essential to separate immigrant and native fertility patterns for Hispanic women, though immigrants also shape conclusions about childbearing differentials between Black and White women. As the ethno-racial group with the largest foreign-born share, it is also essential to consider the link between nativity and childbearing among Asian women, using alternative data sources such as the Current Population Survey (https://www.census.gov/programs-surveys/cps.html). We consider this an exciting area for future research.

## Figures and Tables

**Table 1:** Number of women by race and ethnicity across waves of the National Survey of Family Growth (1995–2017) and size of the analytical sample

Race and ethnicity	National Survey of Family Growth – waves							Total
	1995	2002	2006	2008–2010	2011	2013	2015–2017	
White	6,483	4,139	3,105	3,196	2,584	2,701	2,670	24,878
Black	2,446	1,530	1,201	1,334	1,227	1,193	1,341	10,272
Hispanic	1,553	1,589	1,177	1,546	1,458	1,394	1,218	9,935
Other	365	385	368	352	332	411	325	2,538
Total	10,847	7,643	5,851	6,428	5,601	5,699	5,554	47,623
Excluding ‘Other’								45,085
Analytical sample								44,946

*Note*: The analytical sample results from excluding 139 records with missing information in at least one of the covariates.

**Table 2:** Coeffcients from split-population log-normal models predicting first birth. Standard errors (s.e.) in parenthesis

Panel A: Regression estimates for the timing of first birth				
	Model 1	Model 2	Model 3	Model 4
Race/Ethnicity (ref. White)				
Black	−0.125 (0.004)	−0.126 (0.004)	−0.130 (0.004)	−0.099 (0.004)
Hispanic	−0.071 (0.004)	−0.103 (0.005)	−0.090 (0.005)	−0.053 (0.004)
Nativity (ref. US-born and child-migrant)				
Foreign-born		0.080 (0.007)	0.145 (0.018)	0.088 (0.015)
Interaction terms				
FB * Black			0.049 (0.024)	0.061 (0.020)
FB * Hispanic			−0.090 (0.019)	0.002 (0.016)
Educational attainment (ref. <9 years)				
10–12 years				0.069 (0.005)
13–15 years				0.133 (0.005)
16+				0.310 (0.006)
Intercept	3.316 (0.005)	3.313 (0.005)	3.311 (0.005)	3.152 (0.006)
Shape	−1.410 (0.006)	−1.546 (0.006)	−1.418 (0.006)	−1.559 (0.006)
Panel B: Logistic regression estimates for the proportion childless by age 45				
	Model 1	Model 2	Model 3	Model 4
Race/Ethnicity (ref. White)				
Black	−0.335 (0.042)	−0.328 (0.043)	−0.346 (0.043)	−0.091 (0.044)
Hispanic	−0.821 (0.050)	−0.552 (0.056)	−0.491 (0.056)	−0.277 (0.058)
Nativity (ref. US-born and child-migrant)				
Foreign-born		−0.641 (0.079)	−0.573 (0.198)	−0.608 (0.195)
FB * Black			0.198 (0.272)	0.175 (0.263)
FB * Hispanic			−0.366 (0.221)	0.064 (0.218)
Educational attainment (ref. <9 years)				
10–12 years				0.374 (0.077)
13–15 years				0.919 (0.078)
16+				1.208 (0.081)
Constant	−1.563 (0.045)	−1.415 (0.006)	−1.544 (0.045)	−2.363 (0.084)
N	44,946			

*Note*: Models include controls for survey year and birth cohort. Results not reported.

**Table 3:** Predicted mean age at first birth and proportion childless

	Model 1	Model 2	Model 3	Model 4
**Mean age to 1st birth**				
White	27.6	27.5		
Black	24.3	24.2		
Hispanic	25.7	24.8		
Foreign-born		28.3		
Native and child-migrant				
White			27.4	26.6
Black			24.1	24.1
Hispanic			25.1	25.2
Foreign-born				
White			31.7	29.0
Black			29.2	28.0
Hispanic			26.5	27.6
**Estimated percentage childless by age 40**				
White	17.3	19.5		
Black	13.0	14.9		
Hispanic	8.4	12.3		
Foreign-Born		9.5		
Native and child-migrant				
White			17.6	15.3
Black			13.1	14.1
Hispanic			11.6	12.0
Foreign-born				
White			10.7	8.9
Black			7.6	8.0
Hispanic			6.9	7.4

## Appendix

**Table A-1:** Descriptive statistics for variables included in the multivarite analysis

	Full sample	NH-White			NH-Black			Hispanic		
		All	US-born	Foreign	All	US-born	Foreign	All	US-born	Foreign
Race/Ethnicity										
White	66.3									
Black	15.3									
Hispanic	18.5									
Nativity										
Foreign-born	7.9	2.2			6.4			29.9		
Educational attainment (ref. <9 years)										
<=9 years	12.4	7.5	7.7	1.6	9.5	9.8	5.6	23.3	14.4	44.4
10–12 years	39.7	33.3	33.6	21.6	44.5	45.4	30.0	39.5	43.0	31.3
13–15 years	26.4	27.7	27.8	24.1	29.1	28.9	31.0	24.3	29.2	12.7
16+	21.5	31.5	31.0	52.7	17.0	15.8	33.3	12.8	13.4	11.6
Survey year										
1995	23.7									
2002	16.4									
2006	8.2									
2008	8.1									
2011	16.0									
2013	16.1									
2015	11.5									
Cohort										
1950–1960	18.4									
1960–1970	30.5									
1970–1980	26.8									
1980–1990	17.3									
1990+	25.8									
Unweighted N.	44,946	24,848	24,352	496	10,189	9,694	495	9,909	7,015	2,894
